# Vasorelaxant and Antioxidant Effects of *Aframomum pruinosum* Gagnep. (Zingiberaceae) Seed Extracts May Mediate Their Cardioprotective Activity against Isoproterenol-Induced Myocardial Infarction

**DOI:** 10.1155/2022/7257448

**Published:** 2022-02-10

**Authors:** Elvine Pami Nguelefack-Mbuyo, Florence Nokam, Nidele Lonla Tchinda, Ariane Falone Goumtsa, Nole Tsabang, Télesphore Benoît Nguelefack

**Affiliations:** ^1^University of Dschang, Faculty of Science, Research Unit of Animal Physiology and Phytopharmacology, P.O. Box 67, Dschang, Cameroon; ^2^Higher Institute of Environment Sciences, P.O. Box 16317, Yaounde, Cameroon

## Abstract

*Aframomum pruinosum* seeds are traditionally used in Cameroon to treat cardiac palpitations. The present work evaluates the cardioprotective effects of the aqueous (AE) and ethanolic (EE) extracts from *A. pruinosum* seeds against isoproterenol-induced myocardial infarction. Male Wistar rats were pretreated for 14 days with AE or EE at doses of 75 and 150 mg/kg/day or propranolol (10 mg/kg/day). On days 15 and 16, they were injected subcutaneously with isoproterenol (85 mg/kg/day). Blood pressure and heart rate were weekly recorded by tail-cuff plethysmography during pretreatment and 24 hours after the second dose of isoproterenol. At the end of the treatment period, serum Lactate Dehydrogenase (LDH), Alanine Aminotransferase (ALT), Aspartate Aminotransferase (AST), cardiac nitric oxide (NO), myeloperoxidase (MPO), and oxidative stress parameters (SOD, catalase, MDA, and GSH) were assayed. Sections of left ventricle tissue were subjected to histological analysis. The vasorelaxant effects of cumulative concentrations of AE or EE (3–300 *µ*g/mL) were evaluated on intact or endothelium-denuded isolated aorta rings precontracted with noradrenaline (1 *µ*M). The vasorelaxant effects of the plant extracts were also tested in the presence of N^*ω*^-nitro-L-arginine methyl ester (L-NAME; 100 *µ*M). AE and EE significantly prevented blood pressure decrease and heart rate increase elicited by isoproterenol. Both plant extracts inhibited the increase in ALT, AST, NO, and MPO but did not prevent LDH surge. Oxidative stress parameters were improved following *A. pruinosum* pretreatment. AE and EE highly reduced cardiomyocyte necrosis and fibrosis but did not prevent leukocyte infiltration. Both extracts induced a concentration-dependent vasorelaxation that was significantly inhibited by the destruction of the endothelium and by L-NAME. Extracts of *A. pruinosum* exhibited cardioprotective effects, and EE was the most active. The cardioprotective effects of *A. pruinosum* extracts could be ascribed to their antioxidant, antinecrotic, and endothelium-dependent vasorelaxant effects.

## 1. Introduction

Myocardial infarction (MI), commonly known as heart attack, represents a true emergency condition. It is defined as cardiomyocyte necrosis due to prolonged myocardial ischemia as a result of an imbalance between coronary blood supply and myocardial demand [1].

MI is one of the leading causes of arrhythmia, heart failure, and sudden death worldwide. Its prevalence is age-specific and ranges from 0.06% of men <45 years of age to 12.08% of those ≥75 years old [2]. Population case-fatality also increased with age, from 19% (in 35 to 64 years old) to 84% (in 85 to 94 years old) [3]. Despite the increasing burden of cardiovascular diseases in low- and middle-income countries, data concerning MI prevalence in sub-Saharan African are scarce. However, the prevalence of acute myocardial infarction ranged from 0.1 to 10.4% [4]. With a 30.9% prevalence of arterial hypertension [5], which is a risk factor for MI, one could think that many people in Cameroon suffer from MI.

Isoproterenol (ISO) is widely used as an experimental tool for the study of mechanisms underlying MI or to test the efficacy of potential drugs against MI. ISO-induced MI is characterized by physiological, biochemical, and structural changes similar to those observed in humans [6]. Myocardial hyperfunction due to increased chronotropism and inotropism has been evoked as a mechanism underlying MI elicited by high dose ISO [6]. Moreover, ISO-induced MI is associated with the development of oxidative stress and inflammation [1, 7].

To avoid excessive cardiomyocyte injury, reperfusion techniques such as fibrinolysis and primary percutaneous coronary intervention [8, 9] must be urgently put in place to save patient life. Although these therapies have shown their efficacy, they are associated with a second wave of cardiomyocyte injury due to restoration of coronary flow [9], and also, they have been associated with a high risk of reinfarction occurrence [10]. This situation prompts a need to find a novel effective cardioprotective agent.

*Aframomum pruinosum* Gagnep. (Zingiberaceae) seeds are widely used in some regions of Cameroon for women infertility, respiratory tract disorders, and cardiac palpitations, and they are used as a tranquilizer [11–13]. A recent pharmacological study evidenced the antiulcerogenic effects of *A. pruinosum* seeds (Mabeku et al.) [14]. In addition to this, we demonstrated in a previous study the blood pressure lowering effects and the in vitro antioxidant activity of *A. pruinosum* seeds (data not shown). Since the increase in chronotropism and inotropism as seen in the ISO model further leads to increased workload to the heart, we hypothesized that *A. pruinosum* through its antihypertensive and antioxidant effects may counteract the deleterious effects of ISO by reducing the cardiac workload and by stimulating the antioxidant defense of the organism. The present study was, therefore, designed to evaluate the cardioprotective effects of the aqueous and ethanolic extracts from the seeds of *A. pruinosum* against ISO-induced myocardial infarction.

## 2. Materials and Methods

### 2.1. Reagents

ISO, hexadecyltrimethylammonium, trichloroacetic acid, carbachol, butylated hydroxytoluene, D-glucose, gallic acid, quercetin, thiobarbituric acid, and noradrenaline were purchased from Sigma-Aldrich (Germany). Propranolol was purchased from Teva Santé (France). DMSO, EDTA, NADH, Folin–Ciocalteu reagent, and sodium pyruvate were from Carl-Roth (Kalshur, Germany). L-NAME and glibenclamide were bought from Enzo Life Sciences (Lausen, Switzerland). NaHCO_3_ was purchased from Riedel‐de Haën AG. KH_2_PO_4,_ KCl, CaCl_2_, MgSO_4,_ NaCl, and orthophosphoric acid were bought from BDH (chemicals Ltd Poole England).

### 2.2. Animals

Male Wistar rats aged 3 to 4 months and weighing 200–250 g were used in this study. They were bred in our animal house (Laboratory of Animal Physiology and Phytopharmacology, University of Dschang) in plastic cages. Rats were maintained at a room temperature of 23 ± 2°C under natural light/dark cycle (∼12/12 h). They were supplied with standard laboratory chow and tap water ad libitum. All animals' procedures were done following the standard ethical guidelines for animal use and care as described by the law 2010/63/EU of the European Parliament and of the Council of 22 September 2010 on the protection of animals used for scientific purposes [15] and approved by the institutional ethic committee 025/13/304/FSa.

### 2.3. Plant Collection and Extraction

Fresh seeds of *A. pruinosum* were bought from a local producer at Banguè in the municipality of Yokadouma, East Cameroon region, in August 2017. The plant specimen was authenticated at the national herbarium of Cameroon in comparison with an existing voucher specimen 45393/HNC. The fresh seeds were shade-dried, separated from their sheath, and ground into a fine powder. Three hundred grams of the powder obtained was macerated in 2.25 L sterile distilled water for 24 h. The macerate was filtered, and the filtrate was kept at 4°C. The residue was again macerated in 2.25 L sterile distilled water for another 24 h. Both filtrates were mixed and evaporated in an AISET YLD-2000 ventilated and temperature-controlled oven at 40°C. The extraction procedure yielded 9.21 g aqueous extract.

The ethanolic extract was obtained by macerating 300 g of *A. pruinosum* seeds powder in 2.25 L ethanol for 72 h, with occasional shaking. After filtration, the residue was again macerated in 2.25 L ethanol for 24 h. Both filtrates obtained following this procedure were mixed and concentrated under reduced pressure using a rotary evaporator at 65°C and further in a ventilated and temperature-controlled oven set at 40°C for complete solvent evaporation. This process yielded 14.88 g ethanolic extract.

### 2.4. Phytochemical Analysis

The phytochemical analysis of the two extracts (aqueous extract and ethanolic extract) was performed with both qualitative and quantitative approaches. Qualitative tests for alkaloids, saponins, glycosides, and reducing sugars were performed according to the methods described by Okoha et al. [16]. The quantitative determination of total phenolic compounds was assessed using the Folin-Ciocalteu method [17], while the total flavonoids content was estimated as previously described [18].

### 2.5. Evaluation of the Cardioprotective Effects of *Aframomum pruinosum*

Rats were randomly assigned into 7 groups of 9 rats each except the propranolol group (*n* = 8). Before the induction of myocardial infarction, they were pretreated for two consecutive weeks as follows: the naïve group received per os 1 mL/100 g BW distilled water, the ISO group was orally administered 5% dimethyl sulfoxide (DMSO) solution, group 3 received 10 mg/kg propranolol, and groups 4 and 5 received the aqueous extract of *A. pruinosum* at doses of 75 mg/kg and 150 mg/kg, respectively. Groups 6 and 7 were administered the ethanolic extract at doses of 75 mg/kg and 150 mg/kg, respectively. The aqueous extract was dissolved in distilled water, while the ethanolic extract was prepared in 5% DMSO. Propranolol and plant extracts were given orally. The doses of extract were calculated as recommended by Nair and Jacob [19]. Briefly, the equivalent daily dose of *A. pruinosum* traditionally ingested was found to be 12.1 mg/kg for an adult average weight of 60 kg. This dose was multiplied by 6.2, which is the Km ratio used to convert rat dose to human dose. The dose obtained for rat was 75.02 mg/kg/day. The other dose was obtained by multiplying 75 by 2.

During the pretreatment period, blood pressure and heart rate were indirectly monitored weekly using noninvasive tail-cuff plethysmography (IITC Life Science MRBP tail-cuff blood pressure system). On days 15 and 16, concomitantly with the oral administration of the plant extracts or propranolol, ISO (85 mg/kg/day) was subcutaneously injected to all groups except the naïve group, which was subcutaneously administered with sterile normal saline. On day 17, blood pressure and heart rate were measured again, and after that, animals were anesthetized with sodium thiopental (50 mg/kg; i.p). Blood was collected via the abdominal aorta and centrifuged at 1160 × g for 15 min. The serum was collected and used immediately for the quantification of markers of tissue injury, namely, lactate dehydrogenase (LDH), alanine aminotransferase (ALT), and aspartate aminotransferase (AST). The heart was harvested, washed with normal saline solution, wiped, and weighed. In some cases, the left ventricle was dissected from the right ventricle, weighted, and stored at −20°C for further analysis of some oxidative stress and inflammatory markers, while other hearts were fixed in 10% buffered formaldehyde and used for histological analysis.

### 2.6. Assessment of Injury Markers

Kinetic determination of LDH activity was carried out using a modified method of la Société Française de Biologie Clinique [20]. The reagent mixture was composed of 80 mM tris-HCl buffer (pH = 7.2), sodium pyruvate (1.6 mM), NADH (0.2 mM), and NaCl (200 mM). Briefly, 10 *µ*L of serum sample was mixed with 300 *µ*L of the reagent mixture at 25°C. The decrease in absorbance at 340 nm following NADH oxidation was measured 30 sec after the initiation of the reaction and 1, 2, and 3 min after, using a microplate reader (Multiskan FC, Thermo Fisher). LDH activity was calculated using the following formula:

(1) $$\begin{array}{c} \text{LDH }\left(\text{U}/\text{L}\right)=\Delta \text{DO}/\text{min}\times 4921. \end{array}$$

ALT and AST were determined using commercial kits (Sigma, Hungary) as indicated by the manufacturer.

### 2.7. Assessment of Inflammatory Markers

For the assessment of MPO activity, a 10% left ventricle homogenate was prepared in ice-cold phosphate buffer (50 mM; pH = 5.5) and centrifuged at 9500 × g for 15 min at 4°C. The supernatant was discarded, and the pellet was suspended in ice-cold 0.5% hexadecyltrimethylammonium prepared in 50 mM phosphate buffer (pH = 5.5) to obtain a 10% homogenate. It was then subjected to one cycle of freezing and thawing and again centrifuged at 9503 × g for another 15 min. The MPO activity was assayed in the supernatant according to the method of Krawisz et al. [21]. The change in optical density was read at 450 nm.

The amount of nitric oxide (NO) present in the cardiac homogenate was quantified as previously described [22]. The intensity of the chromatophore formed was read at 540 nm.

### 2.8. Determination of Oxidative Stress Markers

For the evaluation of oxidative stress markers, the left ventricle was homogenized in 0.1 M ice-cold phosphate buffer, pH 7.4 containing 1 mM EDTA, and 0.005% butylated hydroxytoluene [23]. Homogenates were prepared using a potter Elvehjem homogenizer so as to obtain 10%. After centrifugation at 9500 × g for 15 min at 4°C (Loncare TGL-16M refrigerated centrifuge, China), the supernatant was collected and used for the determination of total proteins, malondialdehyde (MDA), catalase (CAT), superoxide dismutase (SOD), and reduced glutathione (GSH).

MDA was quantified as described by Uchiyama and Mihara [24] with slight modifications. One hundred microliters of sample was mixed with 500 *µ*L of 1% orthophosphoric acid and 100 *µ*L of 0.67% thiobarbituric acid prepared in 1% trichloroacetic acid. The mixture was boiled for 15 min and cooled on an ice bath and centrifuged at 850 × g for 10 min. The supernatant was collected, and the optical density was read at 532 nm. The amount of MDA was calculated using the following molar extinction coefficient: 1.56 × 10^5^ M^−1 ^cm^−1^.

CAT activity was evaluated according to the method of Sinha et al. [25]. A calibration curve for hydrogen peroxide (H_2_O_2_) was constructed, and optical densities were read at 570 nm. CAT activity was expressed as the amount of H_2_O_2_ decomposed/min/Gram proteins.

The method of Misra and Fridovich [26] was used for the determination of tissue SOD, while the spectrophotometric method described by Sehirli et al. [27] was used to quantify GSH. Optical densities were read at 412 nm, and the amount of GSH was calculated using 1.36 × 10^4^ M^−1^.Cm^−1^ as molar extinction coefficient.

### 2.9. Histopathological Assessment

In each treatment group, the heart of 3 rats was collected and fixed in 10% buffered formaldehyde. The tissue was embedded in paraffin, cut transversely into sections of 5 *µ*m thickness, and stained with hematoxylin and eosin (H & E). The stained sections were examined and photographed under a DN-107T light microscope for myocardial necrosis and inflammatory cell infiltration. Three microphotographs were made and scored from each animal. The mean of the three scores was used as individual data. Myocardial lesion scoring was performed as described by Eltobshy et al. [28]. Histopathological findings were classified as (0) no changes; (1) mild damage: heart tissues show focal myocardial damage or small multifocal degeneration with slight degree of inflammatory cell infiltrate; (2) moderate damage: heart tissues show extensive myofibrillary degeneration and/or diffuse inflammatory cell infiltrate; and (3) marked damage: heart tissues show marked diffuse necrosis with diffuse inflammatory cell infiltrates.

### 2.10. Evaluation of the Vasorelaxant Effects of *Aframomum pruinosum* Extracts

Aortic rings were prepared as previously described [29]. Animals were sacrificed by cervical dislocation and exsanguinated. The thoracic aorta was rapidly removed and placed in a 37°C physiological Krebs solution of the following composition in mM: NaCl 118, KCl 4.7, CaCl_2_ 2.5, MgSO_4_ 1.2, KH_2_PO_4_ 1.2, NaHCO_3_ 25, glucose 11.1, pH 7.4. The aorta was cleaned of fats and connective tissue and rings of 3 to 4 mm long were mounted in a 10 mL organ chamber filled with Krebs solution at 37°C and continuously aerated. Aorta rings were stretched with a passive tension of 1 g, and changes in tension were recorded with an isometric force-displacement transducer (MDE, Heidelberg) connected to a computer installed with a SPELL Advanced Kymograph data Acquisition software (MDE Heidelberg, Germany). The ring vessel was allowed to equilibrate for 60 min, during which the organ was washed every 15 min. Before the beginning of the experiment, the functional integrity of the endothelium was verified by contracting the vessel with 1 *µ*M noradrenaline (NA) and relaxed with 10^−5^ M carbachol. The presence of a functional endothelium was confirmed by a relaxation of more than 60%. In some experiments, the endothelium was mechanically destroyed by gently rubbing the inside lumen of the vessel with a fine catheter. The endothelium was considered destroyed when carbachol failed to relax vessels pre-contracted with NA. The effects of cumulative concentrations (3, 10, 30,100, and 300 *µ*g/mL) of the aqueous and ethanolic extract of *A. pruinosum* or carbachol (10^−8^, 10^−7^, 10^−6^, 10^−5^, and 10^−4^ M) were evaluated on intact or endothelium-denuded aortic rings precontracted with NA (1 *µ*M) in presence or absence of N^*ω*^-nitro-L-arginine methyl ester (L-NAME; 100 *µ*M).

### 2.11. Statistical Analysis

Statistical analysis was performed using GraphPad Prism 8.4. All the results were expressed as means ± standard error of the mean. For single time point data collection (heart weight, LDH, ALT, AST, proteins, MDA, CAT, SOD, and GSH), data were analyzed with one-way ANOVA followed by Tukey post hoc test. For repeated measures (blood pressure and heart rate), data were analyzed with two-way ANOVA with repeated measures followed by Bonferroni post hoc test. For data concerning the vasodilation experiment, EC_50_ values were obtained after logarithmic transformation of the concentration used by the nonlinear regression method. Differences between means were considered significant at *p* < 0.05.

## 3. Results

### 3.1. Phytochemical Analysis

The phytochemical analysis of the aqueous and ethanolic seed extracts of *A. pruinosum* showed the presence of saponins, glycosides, reducing sugars, phenols, and flavonoids in both extracts, while alkaloids are absent. Saponins, glycosides, total phenolic compounds, and flavonoids were more concentrated in the ethanolic extract compared to the aqueous extract (Table 1).

### 3.2. Effects of *Aframomum pruinosum* Seeds on Blood Pressure

**Figure 1:**
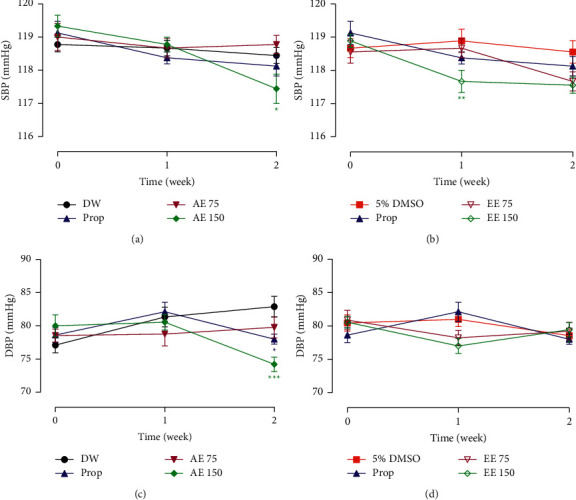
Oral administration of the aqueous (AE) and ethanolic extracts (EE) of *Aframomum pruinosum* seeds reduced systolic (SBP), panels (a) and (b) and diastolic blood pressure (DBP), panels (c) and (d) in normal rat. Data are expressed as mean ± SEM (*n* = 8–9). ^*∗*^*p* < 0.05; ^∗∗^*p* < 0.01; ^∗∗∗^*p* < 0.001 statistically different compared to distilled water (DW) or 5% DMSO. Numbers in the legend refer to the dose in mg/kg. Prop: propranolol.

As shown in Figures 1(a) and 1(b), the administration of the aqueous or the ethanolic extract at the dose of 150 mg/kg induced a small but significant decrease in systolic blood pressure (SBP) ( *p* < 0.05 and *p* < 0.01). The aqueous extract at the dose of 150 mg/kg also significantly (*p* < 0.001) reduced the diastolic blood pressure (DBP) when compared to animals that received distilled water (Figure 1(c)). The ethanolic extract at all doses used did not affect the DBP (*p* < 0.05) of healthy rats when considering the initial value (Figure 1(d)).

**Figure 2:**
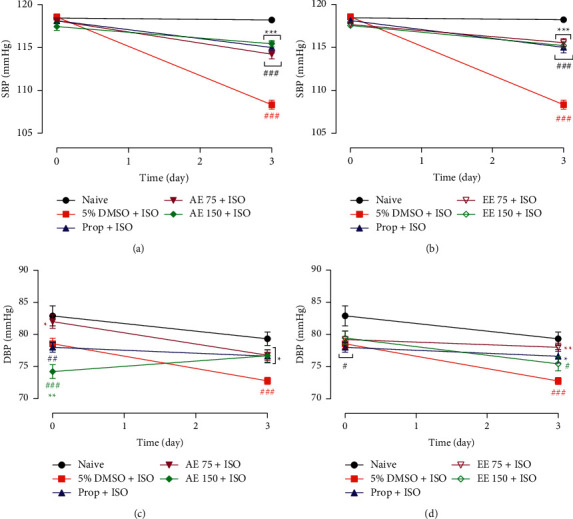
Oral administration of the aqueous (AE) and ethanolic extracts (EE) of *Aframomum pruinosum* seeds prevented the reduction in systolic (SBP), panels (a) and (b) and diastolic blood pressure (DBP), panels (c) and (d) induced by isoproterenol (ISO) in rat. Animals were treated for two weeks before induction of myocardial infarction with isoproterenol (ISO, 85 mg/kg). Data are expressed as mean ± SEM (*n* = 8–9). ^#^*p* < 0.05; ^##^*p* < 0.01; ^###^*p* < 0.001 statistically different compared to naive. ^*∗*^*p* < 0.05; ^∗∗^*p* < 0.01, statistically different compared to 5% DMSO + ISO. Numbers in the legend refer to the dose in mg/kg. Prop: propranolol.

The administration of ISO alone significantly lowered SBP and DBP (*p* < 0.001). The SBP dropped from 118.60 ± 0.34 mmHg to 108.30 ± 0.53 mmHg, while DBP reduced from 78.55 ± 0.49 to 72.77 ± 0.13 mmHg. The concomitant administration of ISO with the plant extracts at all doses or propranolol significantly (*p* < 0.05 to 0.001) inhibited this drop in SBP and DBP elicited by ISO (Figure 2).

### 3.3. Effects of *Aframomum pruinosum* Seeds on Heart Rate

**Figure 3:**
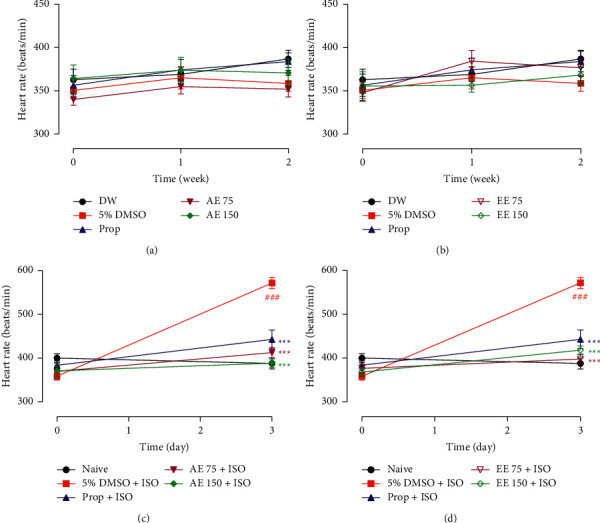
Oral administration of the aqueous (AE) and ethanolic extracts (EE) of *Aframomum pruinosum s*eeds did not affect the heart rate (panels (a) and (b)) but significantly prevented the tachycardia (panels (c) and (d)) induced by isoproterenol (ISO) in rats. Animals were treated for two weeks before induction of myocardial infarction with isoproterenol (ISO, 85 mg/kg). Data are expressed as mean ± SEM (*n* = 8–9). ^###^*p* < 0.001 statistically different compared to distilled water (DW) or naive. ^∗∗∗^*p* < 0.001 statistically different compared to 5% DMSO + ISO. Numbers in the legend refer to the dose in mg/kg. Prop: propranolol.

As observed in Figure 3, the plant extracts did not affect the heart rate of healthy rats. The administration of ISO caused a 1.6-fold increase in heart rate (*p* < 0.001). The heart rate of animals that received only ISO changed from 358.60 ± 9.11 bpm to 571.80 ± 12.95 bpm. This effect was completely inhibited (*p* < 0.001) by plant extracts and by propranolol.

### 3.4. Effects of *Aframomum pruinosum* Seeds on Cardiac Mass

**Figure 4:**
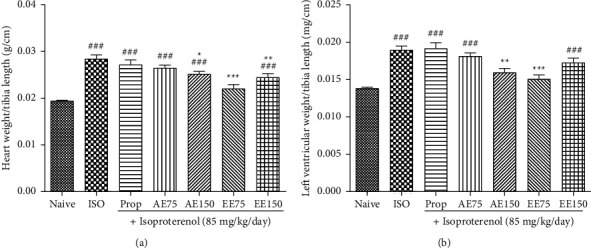
Isoproterenol significantly increased heart (panel (a) and left ventricular (panel (b) relative weight that was prevented by pretreatment with the aqueous (AE) and ethanolic extract (EE) of *Aframomum pruinosum* seeds, administered orally. Animals were treated for two weeks before induction of myocardial infarction with isoproterenol. Data are expressed as mean ± SEM (*n* = 8–9). ^###^*p* < 0.001 statistically different compared to naive. ^*∗*^*p* < 0.05; ^∗∗^*p* < 0.01; ^∗∗∗^*p* < 0.001 statistically different compared to ISO. Numbers refer to the dose in mg/kg.

The induction of myocardial infarction using ISO resulted in cardiac hypertrophy compared to the naïve control group (Figure 4). The plant extracts and mostly the ethanolic extract at the dose of 75 mg/kg highly prevented both cardiac and left ventricular enlargement as depicted in Figures 4(a) and 4(b) (*p* < 0.001).

### 3.5. Effects of *Aframomum pruinosum* Seeds on Tissue Injury Markers

**Figure 5:**
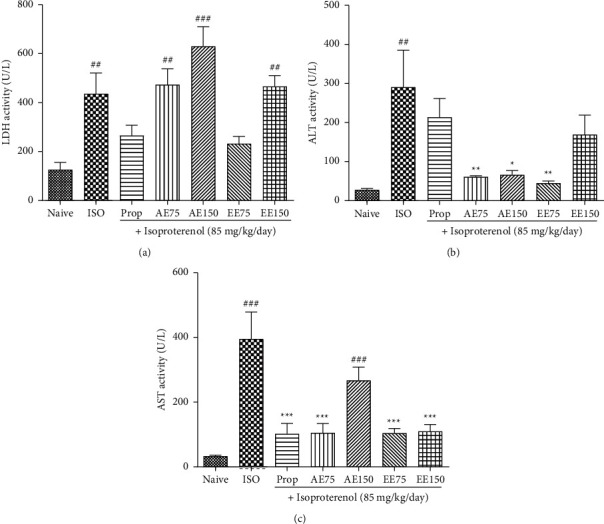
The aqueous (AE) and ethanolic extract (EE) of *Aframomum pruinosum* seeds administered orally prevented the rise in blood lactate dehydrogenase (LDH); panel (a), alanine amino transferase (ALT); panel (b) and aspartate amino transferase (AST); panel (c) following isoproterenol (ISO) induced myocardial infarction. Animals were treated for two weeks before induction of myocardial infarction with isoproterenol. Data are expressed as mean ± SEM (*n* = 6–8). ^##^*p* < 0.01; ^###^*p* < 0.001 statistically different compared to naive. ^*∗*^*p* < 0.05; ^∗∗^*p* < 0.01; ^∗∗∗^*p* < 0.001 statistically different compared to ISO. Numbers refer to the dose in mg/kg.

It was found that the treatment of animals with ISO caused a significant 74.24% increase in LDH levels (*p* < 0.01). Propranolol and ethanolic extract at the dose of 75 mg/kg mitigated the effect of ISO on serum LDH although nonsignificant (*p* < 0.05). In contrast, the aqueous extract at the dose of 150 mg/kg potentiated the effect of ISO (Figure 5(a)).

The serum content in ALT and AST increased drastically following treatment with ISO alone. The serum levels of ALT and AST in the ISO group were respectively, ten and twelve-time higher than those of the naïve group (*p* < 0.01 and 0.001). It was noticed in Figures 5(b) and 5(c) that the plant extracts significantly prevent the rise of both ALT and AST, with the aqueous extract being more efficient on ALT, while the ethanolic extract inhibited more efficiently the increase in AST level. In general, the lower dose of extracts used (75 mg/kg) was more active than the highest dose (150 mg/kg).

### 3.6. Effects of *Aframomum pruinosum* Seeds on Inflammation Markers

**Figure 6:**
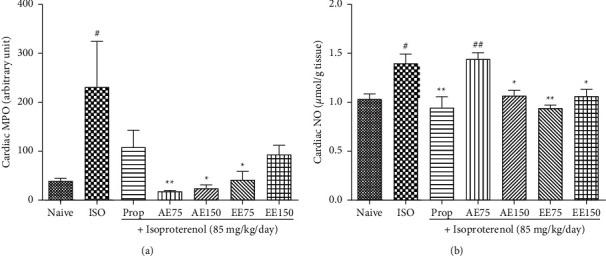
The aqueous (AE) and ethanolic extract (EE) of *Aframomum pruinosum* seeds administered orally prevented the rise in cardiac myeloperoxidase (MPO); panel (a) and nitric oxide (NO); panel (b) content following isoproterenol (ISO) induced myocardial infarction. Animals were treated for two weeks before induction of myocardial infarction with isoproterenol. Data are expressed as mean ± SEM (*n* = 5–6). ^#^*p* < 0.05; ^##^*p* < 0.01; statistically different compared to naive. ^*∗*^*p* < 0.05; ^∗∗^*p* < 0.01; statistically different compared to ISO. Numbers refer to the dose in mg/kg.

From the results depicted in Figure 6(a), it can be noticed that ISO administration significantly (*p* < 0.05) increased cardiac MPO activity. This increase in MPO activity was abolished whatever the dose or type or extract used is, with the aqueous extract at the dose of 75 mg/kg being the most active.

Cardiac NO was significantly augmented in the ISO group (*p* < 0.05). Treatment with plant extracts completely inhibited this augmentation, except the aqueous extract at the dose of 75 mg/kg that fails to circumvent the effect of ISO as illustrated in Figure 6(b).

### 3.7. Effects of *Aframomum pruinosum* Seeds on Oxidative Stress Markers

MDA levels drastically increased (*p* < 0.001) following ISO injection in rat cardiac tissue. This effect was associated with a nonsignificant decrease (*p* > 0.05) in CAT and SOD activity, and a decline in GSH levels. All these alterations elicited by ISO were completely abolished by the plant extracts. They were particularly efficient in reducing the MDA content (*p* < 0.001) and increasing the GSH level, as compared to the ISO group. The administration of the ethanolic extract, especially at the dose of 75 mg/kg, resulted in a greater (*p* < 0.001) CAT and SOD activity than that of both the naïve group that received distilled water only and the disease control group (ISO) (Table 2).

### 3.8. Effects of *Aframomum pruinosum* Seeds Cardiac Histology

**Figure 7:**
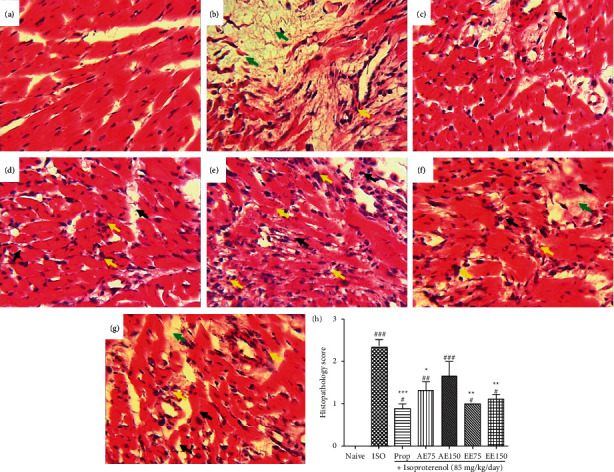
Aqueous and ethanolic extracts from the seeds of *Aframomum pruinosum* protected rat heart against isoproterenol-induced myocardial infarction. Pictures present transverse sections of heart apexes of naïve (a) isoproterenol control (b) isoproterenol + captopril (c) isoproterenol + aqueous extract at the dose of 75 mg/kg (d) or 150 mg/kg (e) and isoproterenol + ethanolic extract at the dose of 75 mg/kg (f) or 150 mg/kg (g) Yellow arrows indicate immune cell infiltration, green arrows indicate fibrosis and black arrows indicate cardiomyocyte necrosis. Slices were stained with hematoxylin & eosin. Magnification × 400.

From the transverse sections of the heart apex (Figure 7), it appears that animals that did not receive ISO (naïve group) presented a well-structured cardiac tissue showing a normal cardiac muscle fibers architecture without necrosis and inflammation. In animals treated with ISO alone, large areas of muscle cell necrosis and fibrosis are observed as well as leukocyte infiltration. In animals treated with either propranolol or *A. pruinosum* extracts followed by ISO injection, the cardiomyocytes necrosis, as well as fibrosis, was greatly reduced. Curiously, the administration of the aqueous extract was associated with a high leucocyte infiltration, while the ethanolic extract, especially at the dose of 150 mg/kg, largely prevented immune cell infiltration. As seen in Figure 7(h), propranolol and the plant extracts, except the aqueous extract at the dose of 150 mg/kg, significantly reduced (*p* < 0.05) ISO-induced myocardial lesions.

### 3.9. Effect of *Aframomum pruinosum* Seeds on Isolated Aortic Rings

**Figure 8:**
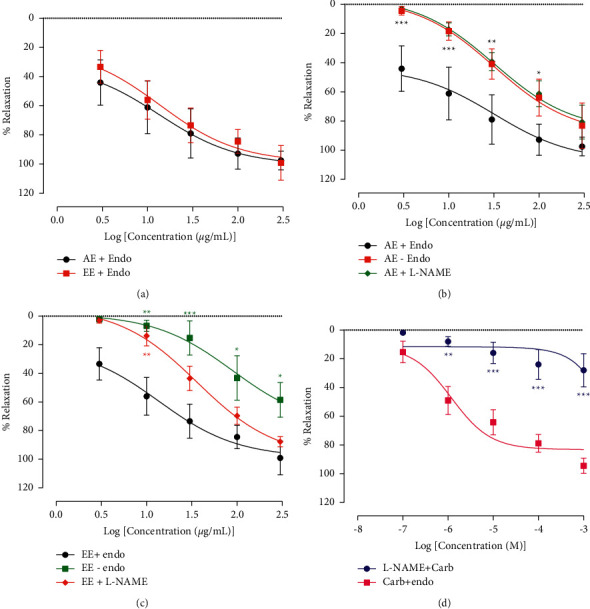
The aqueous (AE) and ethanolic extracts (EE) from *Aframomum pruinosum* seeds induced concentration-dependent vasorelaxation of thoracic aorta rings precontracted with noradrenaline (panel (a)). The effects of the plant extracts were tested in the presence (+endo) or absence (−endo) of a functional endothelium or N^*ω*^-nitro-L-arginine methyl ester (L-NAME); panels (b) and (c). Panel (d) shows the effects of carbachol (Carb) on intact aorta rings and in presence of L-NAME. *n* = 5–6. ^*∗*^*p* < 0.05; ^∗∗^*p* < 0.01; ^∗∗∗^*p* < 0.001 compared to EE + endo or Carb + endo.

Cumulative concentrations of the aqueous and ethanolic extract from *A. pruinosum* seeds induced a concentration-dependent relaxation of aortic rings precontracted with NA. The vasorelaxant activities of both extracts were almost similar, with respective EC_50_ of 13.46 and 13.74 *µ*g/mL (Figure 8(a)). The removal of the endothelium and the preincubation of the aortic ring with L-NAME similarly and significantly (*p* < 0.001) reduced the relaxant effect of the aqueous extract, increasing its EC_50_ from 13.46 *µ*g/mL to 34.84 and 34.42 *µ*g/mL, respectively (Figure 8(b)). Concerning the ethanolic extract, the destruction of the endothelium also significantly (*p* < 0.001) reduced its vasodilating effect, thereby, increasing the EC_50_ value from 13.74 *µ*g/mL to 89.53 *µ*g/mL. In presence of L-NAME, the response to *A. pruinosum* was reduced but to a lesser extent than the removal of the endothelium. The EC_50_ was significantly (*p* < 0.001) increased to 36.16 *µ*g/mL (Figure 8(c)). L-NAME almost completely (*p* < 0.001) inhibited the vasorelaxant effect of carbachol used here as the reference drug (Figure 8(d)).

## 4. Discussion

MI is the most frequent cause of cardiovascular disease-associated mortality [30]. In this study, MI was induced by acute subcutaneous injection of 85 mg/kg ISO for two consecutive days. The results showed ISO-induced hemodynamic changes, cardiomyocyte necrosis, inflammation, and oxidative stress. These alterations were partially or totally prevented by *A. pruinosum* extracts depending on the parameter considered.

ISO is a *β*-adrenergic receptor agonist. When used in high doses, it causes an imbalance between cardiomyocyte demand and supply in oxygen through augmented chronotropism and inotropism [31]. The increase in heart rate observed in this study is in accordance with the chronotropic effect of ISO. ISO-induced increase in heart rate was abolished by the plant extracts suggesting that these plant extracts may possess a negative chronotropic effect. These extracts are more likely to act as propranolol, a beta-receptor blocker, which also did not reduce heart rate but prevented the ISO-induced tachycardia.

The pretreatment of rats with plant extracts at the dose of 150 mg/kg prior to ISO administration induced a significant decrease in blood pressure. This evidenced the blood pressure lowering effects of *A. pruinosum* seeds.

In this study, rats treated with ISO have a significant low value of blood pressure. Decreased blood pressure value is a common feature of ISO-induced myocardial infarction [31–33] and results from the peripheral activation of *β*_2_-adrenergic receptors that reduced peripheral resistances. The administration of plant extracts abrogated this effect, suggesting that they may be beneficial in the management of heart failure and further support the hypothesis of a *β*-adrenergic antagonism as the mechanism of *A. pruinosum* seeds.

It is well known that high doses of ISO result in cardiac hypertrophy [34]. Inflammation of the cardiac tissue and excessive inotropic effects have been evoked as the mechanisms underlying this cardiac hypertrophy [35]. Sections of cardiac apexes of rats from the ISO group showed a marked leukocyte infiltration in the heart tissue, which indicates an inflammatory reaction. Several authors have shown that ISO-induced myocardial infarction was accompanied by an increase in inflammation mediators like interleukin-1, interleukin-6, and tumor necrosis factor-alpha [1, 7, 34, 36], which would be responsible for cardiac edema [7, 31]. In this study, the anti-inflammatory effects of *A. pruinosum* were evaluated through three main parameters: the serum MPO activity, the cardiac nitric oxide level, and the infiltration of inflammatory cells in the cardiac tissue.

MPO is an inflammation marker, which is released following neutrophils degranulation [37, 38], and the level of MPO is an indication of the intensity of the inflammation process. NO is a pleiotropic factor that regulates a variety of functions in the organism. Zhong et al. [32] reported that NO levels were increased in the plasma of rats that were treated with ISO alone. This increase in plasma NO was accompanied by a decreased expression of eNOS and an increased expression of iNOS showing that, in ISO-induced myocardial infarction, elevated NO levels can be considered as a marker of inflammation.

In the present study, high levels of NO and MPO were noticed in the ISO group and the pretreatment of animals with *A. pruinosum* before the induction of myocardial infarction significantly inhibited cardiac hypertrophy and prevented the rise in cardiac NO and MPO. However, these extracts did not greatly limit the infiltration of leukocyte cells into cardiomyocytes. MPO is an inflammatory enzyme, and its release has been associated with cardiac hypertrophy and continuous activation and recruitment of leukocytes to infarcted tissue [39]. It would have been expected that the plant extracts prevent leukocyte infiltration, given the normal level of MPO observed following their administration. This seemingly contradictory result suggests that AE and EE may act either by preventing neutrophils degranulation or by inhibiting MPO activity. The ability of extracts to prevent cardiac hypertrophy could therefore be ascribed to their anti-inflammatory effects especially their action against MPO. Also, the possibility that the plant extracts might probably prevent the myocardial infarction by blocking the inotropic effect of ISO cannot be ruled out.

During acute myocardial infarction, the levels of LDH, AST, and ALT enzymes increased drastically and are used as markers of myocardial cell membrane damage [40]. ISO significantly triggered the leakage of these enzymes, which is histologically characterized by cardiomyocyte necrosis. Most importantly, MPO appears more and more as a reliable marker of myocardial infarction [38, 41], and it is known to promote tissue injuries [42, 43]. In rats treated with the plant extracts, levels of AST, ALT, and MPO were considerably reduced or nearly normal, which evidenced the cardioprotective effect of these plant extracts. These findings were also concordant with the histological analysis, which showed a significant reduction in myocardial necrosis in rats treated with plant extracts.

Apart from its role in inflammation, MPO also triggers oxidative stress [39, 43, 44]. Moreover, the autoxidation of ISO produces free radicals [45], which cause the peroxidation membrane lipids with concomitant formation of MDA [30, 45]. MDA accumulation will, in turn, produce other radicals, and the final result is a decrease in the frontline antioxidant defense, namely, catalase, SOD, and GSH [45]. In the present study, ISO induced an increase in cardiac MDA associated with a decrease in catalase, SOD, and GSH in the heart. This redox imbalance was prevented by plant extracts that reduced the MDA content and increased the GSH level, and in some cases, the catalase activity. These results showed that *A. pruinosum* seeds possess antioxidant activity and that the results observed are not linked to the effect of *A. pruinosum* seeds on MPO release only.

The antioxidant and cardioprotective effects exhibited by the plant extracts might result from the presence of flavonoids and glycosides. It has been reported by Ciumarnean et al. [46] that flavonoids, a class of secondary metabolites, protect myocardial cells from infarction due to their antioxidant effect. Also, Long et al. [47] showed that total paeony glycosides exert cardioprotective effect against ISO-induced myocardial infarction through the reduction of oxidative stress.

One of the main strategies to reduce or prevent MI is to lower the heart workload either by negative inotropic/chronotropic effects or by reducing the post-charge. It was already shown that *A. pruinosum* extracts could prevent the positive inotropic effect of ISO. To determine the effects of these extracts on the postcharge, their vasorelaxant activities were evaluated. The results showed that the aqueous and ethanolic extracts of *A. pruinosum* concentration-dependently relaxed aortic rings precontracted with noradrenaline. A vasorelaxant effect can be achieved by an endothelium-dependent (indirect vasodilators) or a non-endothelium-dependent (direct vasodilators) mechanism. In fact, endothelium-dependent vasorelaxation is mediated by endothelium-derived vasodilator factors, such as endothelium-derived hyperpolarizing factor, prostacyclin (PGI_2_), and NO [48, 49]. In other to get more insight into the vasorelaxant mechanism of the plant extracts, they were tested on aortic rings without endothelium and on aortic rings preincubated with L-NAME, an inhibitor of nitric oxide synthase. The two experimental conditions significantly reduced the vasorelaxant effects of the plant extracts but did not abolish them, suggesting that *A. pruinosum* extracts possess both endothelium-dependent and non-endothelium-dependent effects. The effect of the aqueous extract was similarly reduced by L-NAME and the removal of endothelium, suggesting that the endothelium-dependent effect of the aqueous extract relies on the production of nitric oxide. The removal of the endothelium affected more the activity of the ethanolic extract than the pretreatment with L-NAME. This finding suggests that the endothelium-dependent vasorelaxant effect of this extract does not depend solely on the NO but also on other endothelium factors such as PGI_2_ or endothelium-derived hyperpolarizing factor. However, this requires further evidence.

Despite the fact that this study indicates some mechanisms underlying the cardioprotective effect of *A. pruinosum*, further studies are needed to elucidate the molecular mechanism involved in the cardioprotective effects of the plant extracts.

## 5. Conclusion

Taken all together, the results of this study evidenced the cardioprotective effects of the aqueous and ethanolic extract from the seeds of *A. pruinosum*. The benefic effects of *A. pruinosum* extracts on the heart structure and function could be ascribed to their ability to increase the antioxidant defense mechanism and to reduce cardiac workload. The effects of these plant extracts on cardiac workload may result from the reduction of heart rate and their vasorelaxant activity. The vasorelaxant effects of both extracts are endothelium- and non-endothelium-dependent. The possible mechanisms by which AE and EE exert their cardioprotective effect can be summarized as follows: the extracts exhibit endothelium-dependent and non-endothelium dependent activity, which together with its negative chronotropic effect lead to the decrease in cardiac workload. The antioxidant activity of the plant extracts coupled with decreased cardiac workload contributes to reduced tissue injury.

## Figures and Tables

**Table 1:** Phytochemical constituents of the aqueous and ethanolic extracts from *Aframomum pruinosum s*eeds.

	Aqueous extract	Ethanolic extract
Alkaloids	−	−
Saponins	++	+++
Glycosides	++	+++
Reducing sugars	++	++
Total phenols (mg gallic acid/g sample)	5.56 ± 0.19	6.66 ± 0.10^∗∗^
Total flavonoids (mg quercetin/g sample)	14.52 ± 0.23	19.32 ± 0.17^∗∗∗^

−(absent), ++ (moderately present), +++ (present in great amount). ^∗∗∗^*p* < 0.001 statistically different compared to the aqueous extract.

**Table 2:** The aqueous (AE) and ethanolic extracts (EE) from *Aframomum pruinosum* seeds improved the redox status in the heart of rats subjected to isoproterenol (ISO) induced myocardial infarction.

Experimental groups	SOD (U/mg protein)	CAT (U/mg protein)	MDA (nmole/g tissue)	GSH (nmole/g tissue)
Naive	19.20 ± 2.07	13.67 ± 1.43	10.90 ± 2.00	981.90 ± 83.37
ISO	8.07 ± 0.92	8.62 ± 0.66	24.31 ± 0.92^###^	396.80 ± 26.24^###^
Prop + ISO	44.97 ± 8.98	49.53 ± 9.19^*∗*^	4.17 ± 0.75^∗∗∗^	844.10 ± 94.94^∗∗^
AE 75 + ISO	15.12 ± 1.28	12.46 ± 0.91	4.27 ± 0.56^∗∗∗^	1204.00 ± 103.40^#∗∗∗^
AE 150 + ISO	28.90 ± 7.82	19.08 ± 4.04	10.52 ± 2.92^∗∗∗^	800.20 ± 53.26^∗∗^
EE 75 + ISO	119.50 ± 30.93^###∗∗∗^	107.00 ± 18.86^###∗∗∗^	9.29 ± 2.46^∗∗∗^	647.80 ± 89.23
EE 150 + ISO	42.02 ± 12.32	35.49 ± 7.99	6.55 ± 1.87^∗∗∗^	737.30 ± 37.37^*∗*^

Data are expressed as mean ± SEM (*n* = 5–6). ^##^*p* < 0.05; ^###^*p* < 0.001 statistically different compared to naive. ^*∗*^*p* < 0.05; ^∗∗^*p* < 0.01; ^∗∗∗^*p* < 0.001 statistically different compared to ISO. Numbers refer to the dose in mg/kg.

## Data Availability

The data used to support the findings of this study are available from the corresponding author upon reasonable request.
